# Preservative Influence of Vaccination

**Published:** 1845-08

**Authors:** 


*Preservative Influence of Vaccination.*—M. Bousquet, of Paris, is of opinion, that the efficacy of vaccine virus becomes diminished by its transmission through a number of individuals; and, in support of this opinion, appeals to the fact, that the effects produced by the renovated virus introduced in 1836 were much severer than those which followed the insertion of the old matter. It is, however, a curious fact, that the difference between the vesicle resulting from the new and that from the old virus is not perceptible until the sixth or seventh day, by which time the preservative power of vaccination has been exerted—which is proved by the circumstance that second vaccination after this period does not produce any effect. Nevertheless, from the increased frequency of variolous and varioloid affections of late years, and from the fact that the greater number of those attacked with small pox subsequent to vaccination are adults, M. Bousquet is led to believe that a diminution in the activity of the vaccine virus does result from its repeated transmission through a number of individuals, and that the full preservative power of vaccination does not extend beyond a certain term of years, although its modifying powers continue throughout life. He advocates, therefore, the plan of re-vaccination. M. Gauthier de Claubry, on the other hand, maintains that there does not exist any ratio between the severity of the local effects of vaccination and its preservative power. He considers the severer symptoms which have followed the insertion of matter direct from the cow, merely as a phenomenon attendant upon the naturalization of cow pox in the human subject. When this has been once accomplished, the transmission of the virus through hundreds of individuals in no degree impairs its virtues, or modifies the character of the eruption to which it gives rise. In proof of this, he refers to the fact that, in some places, the same virus has been employed for twenty-five, thirty, or even forty years, and yet the character of the vesicle produced by it has continued unchanged during the whole period. No argument, M. de Claubry maintains, can be drawn from the occurrence of secondary small pox, since this takes place in persons who have had variola in the natural way—which he states to occur in one of every sixty-three of those who have been spontaneously variolated. M. de Claubry asserts, also, that varioloid eruptions were as frequent in the early days of vaccination as they are now, though their variolus character was not then apprehended; and he denies that such eruptions are confined to adults. He, finally, endeavors to weaken the positive evidence in favor of re-vaccination, by showing that varicella, varioloid eruptions, and even variola, have occurred in the Prussian army, among persons who had been vaccinated.

In a recent communication from Dr. Gregory, physician to the Small Pox Hospital of London, ten cases are noticed of death in children, from small pox occurring after vaccination. The ages of these ten children were as follows:—1 of 9 years; 1 of 8; 2 of 6; 1 of 5; 3 of 4; 1 of 3; and one of 6 months. In all these cases, it is stated by Dr. Gregory that the evidence of prior vaccination was such as to justify the Register in recording them as cases of fatal small pox after vaccination. The children were vaccinated in different localities, showing that the source of the vaccine imperfection was not local; while the respectability of the vaccinators, and the appearance of scars in eight of them, forbid the assumption of entire irregularity in the vaccine process.

Now if ten deaths of children, from variola occurring after vaccination, are recorded in one year, is it not fair to presume that a much larger number of cases of varioloid eruption, which terminated favorably, occurred in children during the same period.—*Transac. Coll. Phys. Phila.*

